# iRGD-Targeted Peptide Nanoparticles for Anti-Angiogenic RNAi-Based Therapy of Endometriosis

**DOI:** 10.3390/pharmaceutics15082108

**Published:** 2023-08-09

**Authors:** Anna Egorova, Mariya Petrosyan, Marianna Maretina, Elena Bazian, Iuliia Krylova, Vladislav Baranov, Anton Kiselev

**Affiliations:** 1 Department of Genomic Medicine, D.O. Ott Research Institute of Obstetrics, Gynecology and Reproductology, Mendeleevskaya Line 3, 199034 Saint-Petersburg, Russia; egorova_anna@yahoo.com (A.E.);; 2 Pharmacology Group, D.O. Ott Research Institute of Obstetrics, Gynecology and Reproductology, Mendeleevskaya Line 3, 199034 Saint-Petersburg, Russia; 3 Department of Pathology, Pavlov First Saint-Petersburg State Medical University, L’va Tolstogo Street 6-8, 197022 Saint-Petersburg, Russia

**Keywords:** endometriosis, siRNA delivery, peptide-based carriers, gene therapy, VEGFA, integrins, iRGD, anti-angiogenic therapy

## Abstract

Anti-angiogenic RNAi-based therapy can be considered as a possible strategy for the treatment of endometriosis (EM), which is the most common gynecological disease. Targeted delivery of siRNA therapeutics is a prerequisite for successful treatment without adverse effects. Here we evaluated the RGD1-R6 peptide carrier as a non-viral vehicle for targeted siRNA delivery to endothelial cells in vitro and endometrial implants in vivo. The physicochemical properties of the siRNA complexes, cellular toxicity, and GFP and VEGFA gene silencing efficiency were studied, and anti-angiogenic effects were proved in cellular and animal models. The modification of siRNA complexes with iRGD ligand resulted in a two-fold increase in gene knockdown efficiency and three-fold decrease in endothelial cells’ migration in vitro. Modeling of EM in rats with the autotransplantation of endometrial tissue subcutaneously was carried out. Efficiency of anti-angiogenic EM therapy in vivo by anti-VEGF siRNA/RGD1-R6 complexes was evaluated by the implants’ volume measurement, CD34 immunohistochemical staining, and VEGFA gene expression analysis. We observed a two-fold decrease in endometriotic implants growth and a two-fold decrease in VEGFA gene expression in comparison with saline-treated implants. RNAi-mediated therapeutic effects were comparable with Dienogest treatment efficiency in a rat EM model. Taken together, these findings demonstrate the advantages of RGD1-R6 peptide carrier as a delivery system for RNAi-based therapy of EM.

## 1. Introduction

Endometriosis (EM) is a complex and often debilitating condition that affects millions of women worldwide [1]. It is a chronic and painful inflammatory disease that occurs when endometrial-like tissue grows outside of the uterus, leading to adhesions, scarring, and infertility. The symptoms of EM vary from woman to woman, but typically include pelvic pain and discomfort, heavy menstrual bleeding, and painful intercourse. EM can also cause a range of other symptoms, such as fatigue, bowel and bladder problems, and depression and anxiety [1].

Although the exact cause of EM is still unknown, a growing body of research has shown that angiogenesis, the process of forming new blood vessels, plays a crucial role in the development and progression of the disease [2]. Angiogenesis is a normal process that occurs in the body during wound healing, tissue repair, etc. [3]. However, in EM, angiogenesis is uncontrolled, leading to the growth and survival of endometrial lesions. These heterotopias are responsible for the pain, heavy bleeding, and other symptoms associated with endometriosis [4].

To combat this process, anti-angiogenic therapy has emerged as a potential treatment for EM. This therapy focuses on inhibiting angiogenesis, which is necessary for the growth and survival of the endometrial lesions [2]. Anti-angiogenic therapy works by blocking the activity of vascular endothelial growth factor (VEGFA), a potent angiogenic factor that is up-regulated in endometriotic lesions, promoting the development of new blood vessels that supply the heterotopias with oxygen and nutrients [5]. By targeting VEGFA, anti-angiogenic therapy can reduce the blood supply to endometriotic lesions and prevent their growth. It has been demonstrated that treatment with angiostatic compounds such as anti-human VEGFA antibody, TNP-470, endostatin, and anginex effectively interferes with the maintenance and growth of endometrial lesions in an EM mouse model [6]. However, undesirable side effects can occur when anti-angiogenic drugs are administered systemically, e.g., impairment of fertility [7,8]. Thus, targeted drug delivery is a prerequisite for successful anti-angiogenic therapy without affecting the normal endothelium. The main strategy to achieve specific drug delivery is the exploitation of ligand-mediated active targeting [9]. An attractive target for specific delivery to endothelium of endometrial lesions is ανβ3 integrin. This glycoprotein plays a role in endometrial receptivity and neoangiogenesis during EM development [10,11]. In fact, neoangiogenic processes in endometriosis share common markers with tumor neoangiogenesis, and overexpression of ανβ3 integrin is found in endothelial cells from ectopic endometrium of endometriosis patients [11,12]. It is worth noticing that down-regulated expression of ανβ3 integrin in eutopic endometrium of women with EM compared with fertile controls was reported previously [13]. This discovery provides a basis for making suggestions that eutopic endometrium will not be affected in case of ανβ3 integrin-targeted anti-angiogenic drug delivery in EM patients.

RNAi-based therapy is an emerging approach which carries enormous potential in treating different types of cancers as well as infections and metabolic, autoimmune, and neurological disorders. Small interfering RNA (siRNA) can selectively silence any gene by targeting its expression at the mRNA level [14]. This unique feature was successfully realized in the development of the first-in-class siRNA drugs such as siRNA/lipid nanoparticles Onpattro (patisiran) and GalNAc-siRNA conjugate Gilvaari (givosiran) in recent years [15,16]. Important to notice is that several recent studies confirmed the feasibility of RNAi-based therapy of EM [17,18,19].

However, siRNA. being an anionic macromolecule. poorly crosses cell membranes and has low bioavailability, and thus should be encapsulated into nanoparticles (NPs) for efficient delivery. Various siRNA delivery systems have been studied so far, including lipid NPs, polymeric NPs, peptide NPs, etc. [15,20,21]. In this context, ligand-modified peptide-based carriers are considered as promising vehicles for targeted siRNA and DNA delivery [22]. Previously, we developed CXCR4-ligand modified peptide NPs bearing anti-VEGFA siRNA and demonstrated efficient down-regulation of the gene expression and subsequent anti-angiogenic effects in vitro and in vivo in the EM model [18,23,24]. Our recent study was devoted to the development of iRGD-modified peptide carrier RGD1 as a vehicle for gene therapeutics delivery to αvβ3 integrin-expressing cancer cells [25]. Further, we have demonstrated that a non-covalent combination of RGD1 and arginine-rich cross-linking peptide R6 makes possible formulation of RGD1-R6/DNA polyplexes for efficient transfection of pancreatic carcinoma and uterine leiomyoma cells [26].

Here, we demonstrate the potential of the RGD1-R6 carrier as a delivery system for RNAi-based therapy of EM. We show that the RGD1-R6 peptide carrier can form stable nanoparticles with siRNA, characterize their physicochemical properties, explore their toxic and transfectional properties in GFP-expressing cancer cells and VEGFA-expressing endothelial cells, and study their ability to inhibit endothelial migration. Finally, we demonstrate how RGD1-R6/siRNA nanoparticles exert their biological activity by being able to induce RNAi-mediated VEGFA gene silencing in a surgically induced EM rat model.

## 2. Materials and Methods

### 2.1. Cell Lines and Animals

GFP-expressing human triple-negative breast cancer cells MDA-MB 231 and human endothelial hybridoma EA.hy926 were maintained under mycoplasma-free conditions as described previously [24,27].

Twenty twelve-week-old nonpregnant female Wistar rats weighting 180–250 g (Rappolovo Breeding Center, Saint-Petersburg, Russia) were maintained in the animal facility and fed with water and a standard diet ad libitum. The animals were acclimated for 2 weeks before surgery. A regular 4–5-day estral cycle was mandatory for entering the experimental protocol. The EM modeling was performed in accordance with the Helsinki declaration, and the study was approved by the Ethics Committee of D.O.Ott Research Institute.

### 2.2. Synthesis of Peptide Carriers

RGD1 (R_9_H_4_CRGDRGPDC), RGD0 (R_9_H_4_), and R6 (CHR_6_HC) peptides were synthesized using a solid-phase Boc-chemistry (NPF Verta LLC, Saint-Petersburg, Russia), as described previously [25,28]. The purity of the peptides (90–95%) was determined by HPLC. RGD1 peptide was dissolved in 2 mg quantities in 0.5 mM Hepes buffer (pH 7.5) at 0.1 mg/mL and allowed to stand overnight at room temperature for formation of cyclic iRGD ligand. After cyclization, the peptide was concentrated to 2 mg/mL using a rotor evaporator (Labconco, Kansas City, MO, USA). Design and composition of the RGD1-R6 and RGD0-R6 peptide carriers were reported in our previous study [26]. Briefly, RGD1-R6 and RGD0-R6 carriers were obtained by mixing solutions of RGD1 or RGD0 peptides, respectively, and R6 cross-linking peptide in equimolar quantities before the addition of siRNA.

### 2.3. siRNAs Sequences

siRNAs were synthesized in Syntol JSC, Moscow, Russia.

The sense strand of anti-GFP siRNA 5′-CAA GCU GAC CCU GAA GUU Ctt-3′ and the sense strand of anti-VEGFA siRNA 5′-GCG GAU CAA ACC UCA CCA Att-3′ were described previously [24,27]. A mock siRNA 5′-UUC UCC GAA CGU GUC ACG Utt-3′ was used as a control [23].

### 2.4. Preparation of siRNA Complexes

siRNA/peptide complexes were prepared at N/P ratios in range 0.5–24 in Hepes-buffered mannitol (HBM; 5% *w*/*v* mannitol, 5 mM Hepes, pH 7.5). The appropriate volume of the peptide carrier in concentration 2 mg/mL was added to the siRNA solution (0.05 mg/mL), vortexed, and allowed to stand for 120 min to ensure cross-linking of R6 peptide. The formulation volume was 40 µL per 1 µg of siRNA.

### 2.5. siRNA Binding Assay

Peptide binding to siRNA was monitored using SYBR Green displacement assay as described previously [25]. Quenching of SYBR Green fluorescence was detected using a Wallac 1420D scanning multilabel counter (PerkinElmer Wallac Oy, Turku, Finland). Binding efficiency was calculated as (F − Ff)/(Fb − Ff), where Ff and Fb are the fluorescence intensities of SYBR Green in the absence and presence of siRNA. The unbound siRNA value was taken as 100%.

### 2.6. DNase I and RNase A Protection Assays

Peptide/siRNA complexes were prepared at various N/P ratios in a volume of 8 µL as described above and incubated with 100 ng of RNase A (BioChemica AppliChem, Darmstadt, Germany) for 30 min at 37 °C. RNase A was inactivated by 1% SDS treatment for 5 min at 98 °C. Then, the complexes were treated overnight with trypsin (0.1%) at 37 °C to release siRNA. Thereafter, siRNA was electrophoresed in an AgNO_3_-stained 15% polyacrylamide gel [29]. The integrity of RNA was assessed by comparison with intact siRNA and RNase A treated siRNA, respectively.

### 2.7. Measurement of Size and Zeta-Potential of siRNA Complexes

The peptide/siRNA complexes were prepared as described above in quantities of 5 µg of siRNA per sample and at an N/P ratio of 8/1. The size of the complexes was determined using dynamic light scattering, and the zeta potential was determined with microelectrophoresis. Three independent measurements were performed on a zetasizer NANO ZS (Malvern Instruments, Malvern, UK).

### 2.8. Cytotoxicity Evaluation of siRNA Complexes

The cytotoxicity of siRNA/peptide complexes formed at N/P ratios 1/8, 1/16, and 1/24 was evaluated in MDA-MB-231 and EA.hy926 cell lines in 96-well plates using Alamar blue assay (BioSources International, San Diego, CA, USA) for cell viability after 16 h of incubation as described previously [25]. Briefly, siRNA was used in concentration 200 nM as well as in transfection experiments. The fluorescence of resorufin was recorded on a Wallac 1420D scanning multilabel counter with wavelengths of 544/590 nm. The relative fluorescence intensity was counted according to (F − Ff)/(Fb − Ff) × 100%, where Fb and Ff are the fluorescence intensities in untreated control and without cells, respectively.

### 2.9. siRNA Transfer to MDA-MB-231 Cells and GFP Fluorescence Detection

The siRNA transfections were performed in triplicates in MDA-MB-231 cells stably expressing GFP as reported previously [25]. Two positive controls of siRNA transfer were used. Polyethyleneimine (branched PEI 25 kDa; Sigma-Aldrich, St. Louis, MO, USA) was used at 0.9 mg/mL (pH 7.5) in aqueous stock solution with a charge ratio of PEI/siRNA 8/1. X-tremeGENE transfection reagent (Roche, Mannheim, Germany) was used according to the manufacturer’s protocol. A total of 70 × 10^4^ cells were seeded in 48-well plates and incubated overnight. The transfections were performed in FBS-free and FBS-supplemented medium. The complexes formed at N/P ratios 1/8, 1/16, and 1/24 bearing anti-GFP siRNA or mock siRNA in concentration 200 nM were incubated with cells for 4 h. Then, after 48 h incubation in FBS-supplemented medium, cells were washed by 1× PBS (pH 7.2) and permeabilized with the reporter cell lysis buffer (25 mM Gly-Gly, 15 mM MgSO4, 4 mM EGTA, 1 mM DTT, 1 mM PMSF; pH 7.8). GFP fluorescence was recorded at wavelengths 485/535 nm by means of a Wallac 1420D counter. The fluorescence level was normalized relative to the total protein concentration in each sample determined by Bradford method.

### 2.10. siRNA Transfer to EA.hy926 Cells and Quantitative RT-PCR

VEGFA gene silencing experiments were performed in triplicates in EA.hy926 cells as previously described [24]. Similarly, x-tremeGENE transfection reagent was taken as a control. The cells (75 × 10^4^) were seeded in 24-well plates and incubated overnight. A FBS supplemented cell culture medium was replaced by medium without FBS before the transfection. The siRNA complexes formed at N/P ratios 1/8 and 1/16 were incubated with cells for 4 h. The final concentration of siRNA was 200 nM per well. After incubation in a fully supplemented cell culture medium for 48 h, cells were taken for RNA extraction using Trizol reagent (Qiagen, Hilden, Germany) and quantitative real-time PCR analysis using the following primers: VEGFA F 5′-GAG CTA AAA ATC TTG ACC CAC ATT G-3′, VEGFA R 5′-CAG TAT TCA ACA ATC ACC ATC AGA G-3′; and reference β-actin gene primers F 5′-TGC CGA CAG GAT GCA GAA G-3′, R 5′-GCC GAT CCA CAC GGA GTA CT-3′. The samples were measured three times, and a final result was inferred by averaging the data. The values are presented as means ± S.E.M of the means obtained from three independent experiments. PCR was performed in thermocycler QuantStudio 5 (Thermo, Waltham, MA, USA). Subsequent analysis was performed using QuantStudio Design & Analysis Software version 1.5.1. A similar protocol was used for assessment of VEGFA gene expression in vivo in EM implants. VEGFA gene expression level in vivo is shown relative to the expression level in control animals injected with saline.

### 2.11. Quantitative Measurement of VEGFA Production

EA.hy926 cells were transfected with anti-VEGFA siRNA or mock siRNA as described above. Thereafter, aliquots of cell culture media (50 μL/well) were taken and analyzed for VEGFA concentration by commercially available human VEGFA ELISA EH2VEGF kit in pre-coated 96-well plates using ready-to-use solutions (Thermo Scientific, Rockford, IL, USA). Absorbance values were recorded using Multiscan plus P plate reader (Labsystems, Vantaa, Finland) at wavelength 450 nm, and VEGFA concentrations were calculated according to the standard curve in pg/mL.

### 2.12. Scratch Migration Assay

Assessment of EA.Hy926 cell migration rate was performed as described previously [23]. siRNA complexes were prepared as described above at N/P ratios of 8/1 and 16/1. A total of 16 × 10^4^ cells were seeded in 96-well plates and incubated overnight. Transfection was performed as described above in quadruplicates. siRNA complexes were incubated with cells for 4 h, then cells were washed by fresh media. Then, the cell monolayer was scratched with a 300 mL pipette tip (Biohit Oy, Helsinki, Finland) and the width of scratch (wound area) was photographed using an MIBR microscope (LOMO, Saint-Petersburg, Russia). After incubation in 2.5% FBS-containing medium for the next 24 h, the cells were stained with 0.2% crystal violet solution and photographed. Three random fields were registered. EA.Hy926 cell migration during the scratch repair was analyzed using ImagePro Plus 6.0 software (Media Cybernetics, Bethesda, MD, USA). The number of cells (n) that migrated to the wound area was counted. Cell density (ρ) was counted in the area of 17,000 mm^2^. The relative number of migrated cells was calculated with (n/n’)× (ρ’/ρ), where n’ is number of migrated cells in untreated control and ρ’ is the cell density in the untreated control.

### 2.13. Induction of EM Rat Model and In Vivo siRNA Transfer

Surgical modeling of EM was performed according to previously published protocol with some changes [18,30]. Briefly, two autologous fragments of uterus were transplanted to the outer surface of the abdominal wall of ovariectomized rats. Before the transfection experiments, the implants were left growing for two weeks. Twenty rats were randomly divided into four groups (n = 4–6). Two injections of anti-VEGFA siRNA or mock siRNA in complexes with RGD1-R6 carrier formed at N/P ratio 1/16 were performed in a one-week interval (total amount of siRNA is 10 µg). The rats were anesthetized and then one endometrial implant was injected with siRNA complexes (dose 5 µg) while the contralateral implant was left intact. Endometrial implants in the negative control group were injected with an appropriate volume of saline. The positive control group was daily for two weeks given Dienogest per os in a dose 1 mg/kg as described previously [31]. One week later the injection was repeated and after one more week the rats were sacrificed. Volumes of injected and contralateral implants were measured before and after treatment (L × W × 2 mm^3^), and immunohistochemical and gene expression analyses were carried out.

### 2.14. Immunohistochemical Detection of CD34 Expression

Immunohistochemical CD34 expression analysis was performed as described previously [32]. Briefly, 3–4 µm thick sections were deparaffinized step-wise in xylene and rehydrated in graded solutions of ethanol. Antigen retrieval and immunoperoxidase staining were performed in a fully automated immunohistostainer BOND-MAX (Leica Biosystems, Wetzlar, Germany). Mouse monoclonal primary antibodies were obtained from Abcam UK (CD34 [EP373Y], clone81289, dilution 1:2500). The resulting micropreparations were scanned using a Leica Aperio AT2 slide scanner with subsequent analysis using the Aperio ImageScope software v.6.25 and the ImageJ Plus 6.0 program.

### 2.15. Statistical Analysis

Statistical analysis was carried out using the Student *t*-test and Mann–Whitney U-test with the GraphPad Prism 8 software package (GraphPad Prism Inc., San Diego, CA, USA). Statistical significance was defined as * *p* < 0.05 and ** *p* < 0.01.

## 3. Results and Discussion

RGD1-R6 peptide carrier has been studied recently as efficient pDNA delivery vehicle targeting αvβ3-expressing cells [26]. Herein, we present characterization of RGD1-R6 as vehicle for targeted delivery of anti-angiogenic siRNA to suppress growth of ectopic endometrium-like tissue in vivo.

### 3.1. Evaluation of Physico-Chemical Properties of siRNA Complexes

Physicochemical properties of siRNA complexes are known to affect their performance in vitro and in vivo [33]. siRNA molecules are too short to be condensed by polycations; however, their electrostatic interaction used to result in molecular collapse and formation of siRNA complexes resistant to nucleases [34].

**Figure 1:**
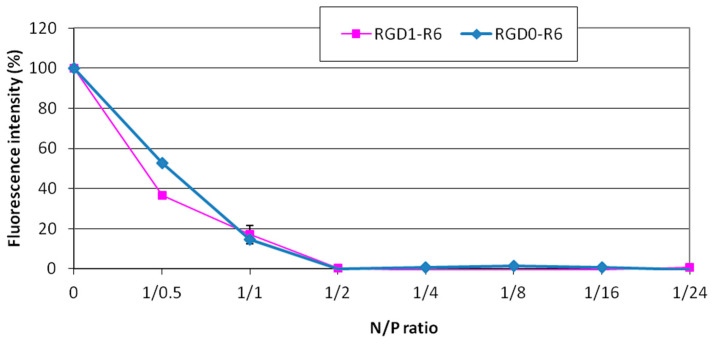
SybrGreen exclusion from complexes of siRNA and RGD1-R6 and RGD0-R carriers. A total of 100% of fluorescence intensity corresponds to free siRNA stained with SybrGreen. The data are shown as the mean ± SD of n = 9 individual samples from three independent experiments.

The siRNA binding with RGD1-R6 and RGD0-R6 carriers was studied using a Sybr Green exclusion assay. As shown in Figure 1, the Sybr Green fluorescence intensity of siRNA complexes decreased substantially at an N/P ratio of 2/1 compared with that of unbound siRNA, taken as 100%. It can be seen that the presence of iRGD as a ligand component of the RGD1-R6 carrier does not affect its binding properties. Thus, it can be supposed that the uncharged ligand does not interact with siRNA and will not affect its affinity to the receptor.

**Figure 2:**
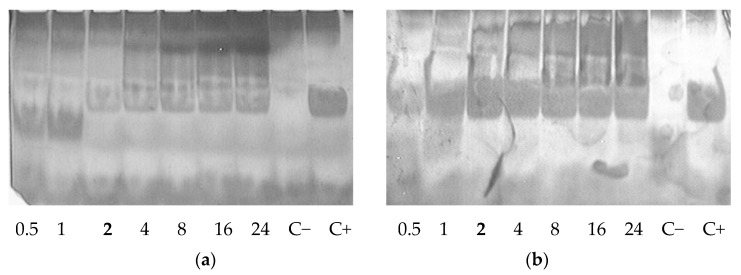
RNase A protective ability of siRNA complexes formed with RGD1-R6 (**a**) and RGD0-R6 (**b**) carriers. C− is siRNA treated with RNase A; C+ is intact siRNA. N/P ratio number in bold indicates full defense from the enzyme degradation.

Sensitivity to nucleases is an important characteristic of non-viral carrier/siRNA complexes [22]. We checked siRNA integrity in the complexes formed at various N/P ratios after treatment with RNase A. Naked siRNA incubated with enzyme was barely detected because of degradation. An increase in the N/P ratio of the complexes resulted in better siRNA protection. Studied carriers were able to protect siRNA from RNase A-mediated degradation at N/P ratio 2/1 (Figure 2). It can be concluded that the carriers have the capacity to provide immediate protection once the siRNA complexes are formed.

Obtained data on physicochemical properties of the presented RGD1-R6 and RGD0-R6-siRNA complexes were compared with a previously published study of RGD1 and RGD0 carriers [25]. We found no difference in siRNA-binding and protective properties between complexes with or without an R6 cross-linking peptide. However, we studied siRNA complexes formed at N/P ratio 8/1 and 16/1 for their size and zeta potential. RGD1-R6/siRNA complexes have size 350.9 ± 3.6 nm and 229.2 ± 4.4 nm and zeta potential 13.1 ± 0.31 mV and 18.8 ± 0.54 mV, respectively. Corresponding RGD0-R6/siRNA complexes have size 339.2 ± 3.9 nm and 249.6 ± 4.5 nm and zeta potential 15.4 ± 0.99 mV and 18.9 ± 0.3 mV, respectively. No significant difference was found between complexes formed at the same charge ratio; however, size and zeta potential values between complexes formed at 8/1 and 16/1 were found significant (*p* < 0.05). If compared with previously studied RGD1 and RGD0 complexes, a significant difference was demonstrated in size. Similar complexes formed at charge ratio 8/1 have size 539 ± 13.55 nm and zeta potential 14.2 ± 0.06 mV, and size 660.9 ± 58.41 nm and zeta potential 17 ± 0.15 mV, respectively [25]. Thus, the positive influence of R6 peptide cross-linking on siRNA complexes size can be demonstrated.

### 3.2. Evaluation of Cytotoxic Properties of siRNA Complexes

**Figure 3:**
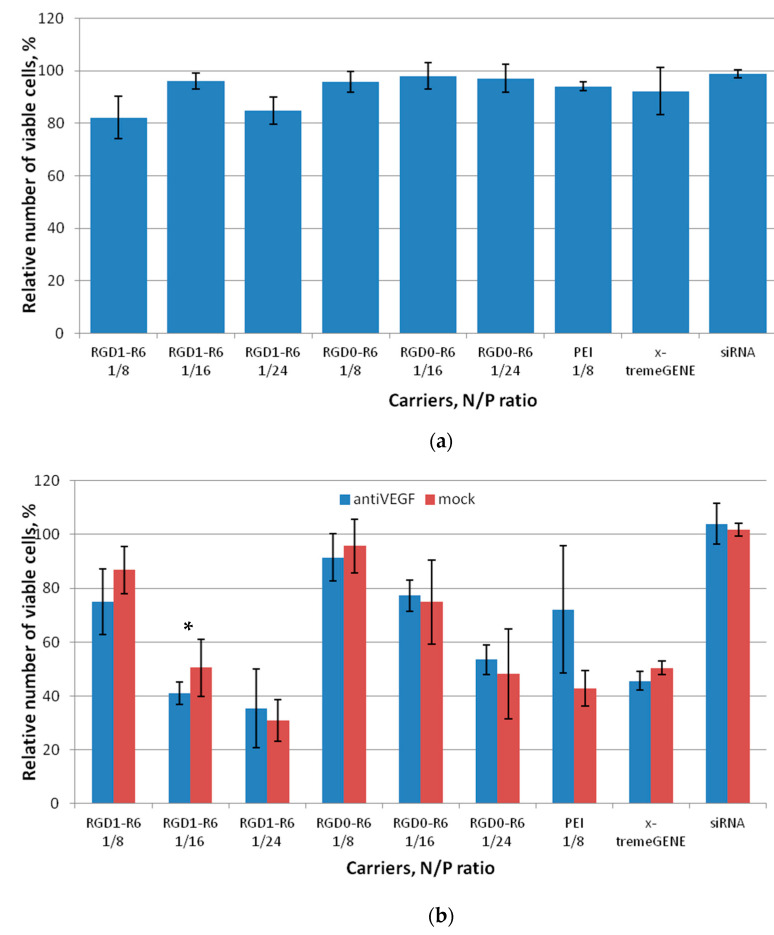
Cytotoxicity evaluation of siRNA complexes in MDA-MB 231 (**a**) and EA.hy926 (**b**) cells by the Alamar blue assay. Values are the mean ± SD of the mean of n = 9 individual samples from three independent experiments. * *p* > 0.05 compared to corresponding RGD0-R6 complexes.

A serious adverse effect of in application of nanoparticles as delivery systems is their cytotoxicity due to the destabilization of membranes and processes through the interaction with extra- and intracellular macromolecules [35]. Herein, we studied cytotoxic properties of siRNA complexes formed at N/P ratios 8/1, 16/1, and 24/1 in cancer cell line MDA-MB 231 and endothelial cell line EA.hy926. Both cell lines express αvβ3 integrins and were chosen for in vitro experiments [36,37]. We demonstrated that RGD1-R6 and RGD0-R6-siRNA complexes do not affect growth of MDA-MB 231, and no difference was found with toxic level of control PEI and x-tremeGENE siRNA complexes (Figure 3a).

In case of VEGFA-expressing endothelial cells we additionally evaluated cytotoxicity of siRNA complexes formed with anti-VEGF siRNA (Figure 3b). We found that complexes formed at a high charge ratio of 24/1 exhibit significant cytotoxicity. Moreover, the EA.hy926 cell line appeared more sensitive to non-viral transfection as both control complexes with PEI and x-tremeGENE reduced the number of viable cells significantly. In contrast, all the siRNA/peptide complexes formed at low charge ratios showed little toxic effects on the endothelial cells. The only exception is RGD1-R6-siRNA complexes at NP ratio 16/1, which demonstrated cytotoxicity level below acceptable 80% viable cells. Despite the fact that VEGFA is important for cellular physiology of endothelial cells, we found no difference between the cytotoxicity level of mock and anti-VEGF siRNA-bearing complexes. The most probable explanation is excessive binding of iRGD-modified complexes with integrins, which play an important role in cell adhesion. It can be supposed that the excessive binding may result in cells detachment [38]. This conclusion can be confirmed by the fact that corresponding RGD0-R6-siRNA complexes are not toxic to the cells.

### 3.3. Evaluation of Gene Expression Silencing Mediated by siRNA Complexes

An MDA-MB-231 cell line stably expressing the GFP gene was used for initial evaluation of RNAi-mediated gene silencing after the delivery of anti-GFP siRNA in complexes with the studied peptide carriers. We were able to demonstrate specific αvβ3-targeted delivery because this cell line expresses αvβ3 integrins on its surface [39]. Specificity of GFP gene expression silencing via RNAi mechanism was proved by using for transfection both anti-GFP and mock siRNAs. Complexes formed at N/P ratios of 8/1, 16/1, and 24/1 were used in this experiment. Naked siRNA transfer was used as negative control.

**Figure 4:**
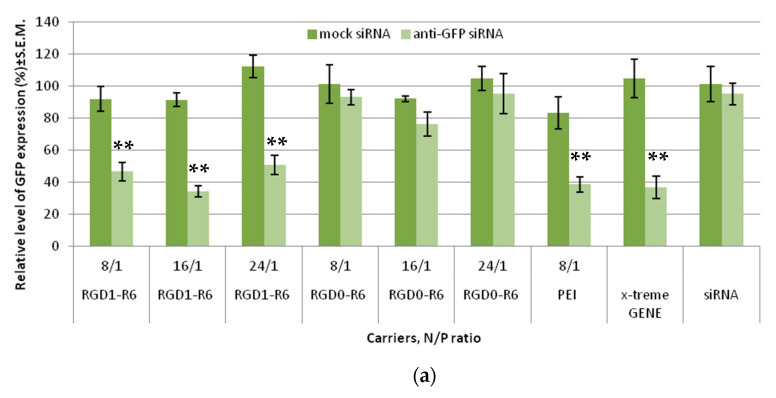

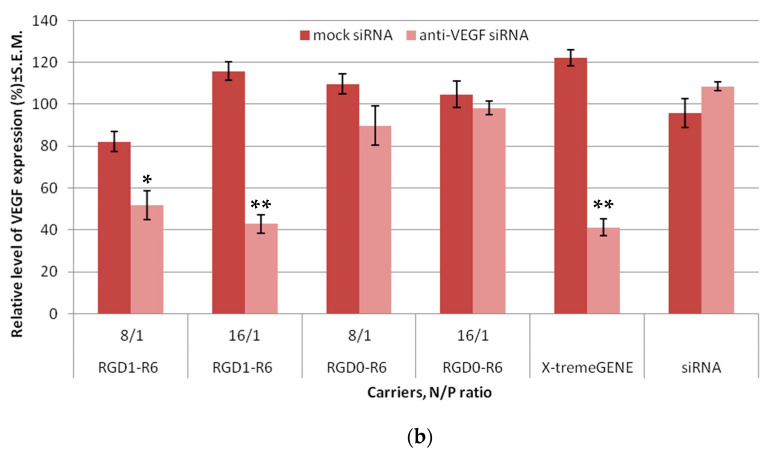
siRNA transfection efficacy evaluation: (**a**) Silencing of GFP expression after treatment of MDA-MB-231 cells with the siRNA complexes. ** *p* < 0.01 when compared with cells treated by corresponding mock siRNA complexes. The data are shown as the mean ± S.E.M. of n = 9 individual samples from three independent experiments. (**b**) Silencing of VEGFA gene expression after treatment of E.A.Hy926 cells with the siRNA complexes. * *p* < 0.05, ** *p* < 0.01 when compared with cells treated by corresponding mock siRNA complexes. The data are shown as the mean ± S.E.M of n = 12 individual samples from three independent experiments.

As shown in Figure 4a, the mock siRNA-bearing complexes cannot downregulate GFP gene expression, which is in sharp contrast with high silencing efficiency of anti-GFP siRNA complexes. This fact undoubtedly confirms that the suppression of GFP gene expression is achieved by means of RNAi but not from the innate complex toxicity. Cell transfection with RGD1-R6/anti-GFP siRNA complexes formed at N/P ratios of 8/1, 16/1, and 24/1 resulted in a decrease in GFP expression to 46.7%, 34.3%, and 50.9% respectively, whereas corresponding RGD0-R6/anti-GFP siRNA complexes downregulated GFP expression only to 93.2%, 76.6%, and 94.4%, respectively. In our opinion, this finding confirms αvβ3-integrin-mediated internalization of RGD1-R6-siRNA complexes to MDA-MB-231 cells and efficient RNAi-mediated GFP gene expression silencing. The silencing efficiency of RGD1-R6-siRNA complexes was comparable with the level of the positive controls. In addition, the transfection was repeated in the presence of fetal bovine serum (FBS) in medium. We found that the presence of FBS negatively influences the transfection efficiency; however, the positive effect of iRGD modification was retained (Figure S1). No decrease in GFP expression was demonstrated after RGD0-R6-mediated delivery of anti-GFP siRNA. However, RGD1-R6/anti-GFP siRNA complexes downregulated GFP expression in the presence of FBS to 70.9%, 70.5%, and 78.6%, respectively. Significant difference (*p* < 0.05) was found with corresponding ligand-free complexes and mock siRNA-bearing complexes. The obtained results are in accordance with a previous study on RGD1-R6-DNA complexes [26].

The study was continued with the EA.Hy926 cell line, which is a well-known cellular model of human endothelium, and represents its main morphological, phenotypical, and functional features [40]. As we proposed RNAi-based suppression of angiogenesis as an approach to EM treatment, it was important to evaluate anti-angiogenic properties of RGD1-R6-siRNA complexes in vitro before conducting experiments on the animal model. siRNA/peptide carrier complexes formed at 8/1 and 16/1 N/P ratios were tested for their ability to suppress VEGFA gene expression. Mock siRNA-bearing complexes were used as a negative control, and their zero efficiency confirmed the RNAi-based mechanism of gene expression silencing. As can be seen in Figure 4b, VEGFA gene expression was decreased by RGD1-R6/anti-VEGFA siRNA complexes up to 51.8% and 42.8%, whereas corresponding RGD0-R6 complexes were unable to suppress expression. The significant difference between efficiency of ligand-modified and corresponding ligand-free complexes (*p* < 0.01) demonstrates the feasibility of αvβ3 integrin-targeted siRNA delivery by means of the RGD1-R6 carrier. Important to note is that the silencing efficiency of RGD1-R6/anti-VEGFA siRNA complexes was equal to the positive control level.

### 3.4. Reduction of VEGFA Protein Secretion Mediated by siRNA Complexes

**Figure 5:**
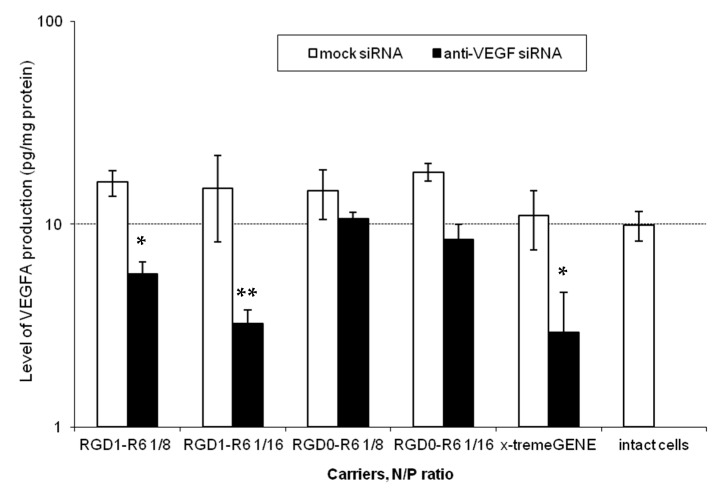
VEGFA protein production (pg/total protein) by EA.Hy926 cells after the treatment with the siRNA complexes. * *p* < 0.05, ** *p* < 0.01 when compared with cells treated by corresponding mock siRNA complexes. The data are shown as the mean ± S.E.M. of n = 9 individual samples from three independent experiments.

Further, to confirm results of successful VEGFA silencing in vitro, we accessed the level of secreted VEGFA protein in the EA.Hy926 cell line. Similar siRNA complexes were taken to the experiment. Results of the quantitative determination of VEGFA protein secretion by the endothelial cells are shown in Figure 5. It can be seen that VEGFA protein is significantly decreased after the treatment with RGD1-R6/anti-VEGFA siRNA complexes, but not if RGD0-R6 carrier or mock siRNA were used. The obtained findings further confirm the conclusions of efficiency and specificity of RGD1-R6-mediated siRNA delivery.

Thus, we demonstrated a two-fold reduction of VEGFA secretion. Additional experiments were conducted to elucidate how the achieved level of reduction can affect angiogenic processes in the endothelial cells.

### 3.5. Inhibition of Endothelial Cells Migration Mediated by siRNA Complexes

The migration of endothelial cells plays a vital role in the formation of new blood vessels [41]. It is characterized by the rapid increase in the number of endothelial cells and their ability to organize themselves into complex three-dimensional structures. These structures then combine with other similar ones to create a network of newly formed blood vessels [42]. Angiogenic factors exert a great influence on the development of new blood vessels, stimulating both the proliferation and differentiation of endothelial cells, and VEGFA is a crucial factor that plays a pivotal role in the process of neoangiogenesis (ref). While migration of endothelial cells is a common occurrence in angiogenesis, it is a rare event in the mature vascular network [43]. This provides a solid foundation for the development of anti-angiogenic treatments for a range of diseases, such as endometriosis.

**Figure 6:**
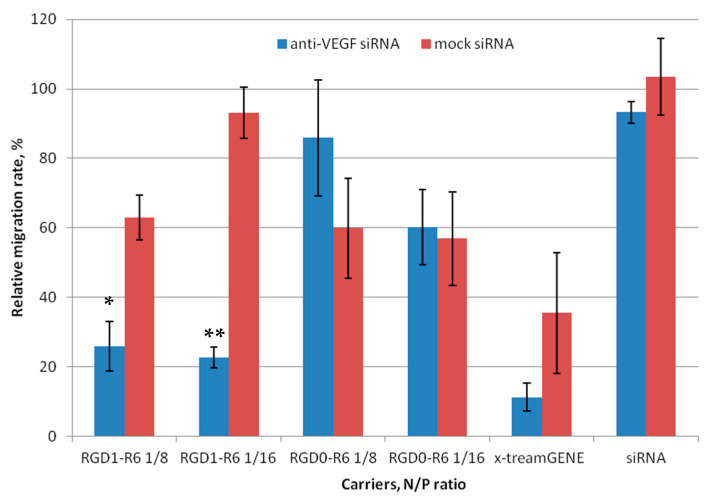
Number of migrated EA.Hy926 cells after the treatment with the siRNA complexes. * *p* < 0.05, ** *p* < 0.01 when compared with cells treated by corresponding mock siRNA complexes. The data are shown as the mean ± S.E.M. of n = 12 individual samples from three independent experiments.

Scratch assays were conducted (Figure 6 and Figure S2) to evaluate the impact of anti-VEGF siRNA complexes on E.A.Hy926 cell migration. The cells were transfected with RGD1-R6 and RGD0-R6 complexes and loaded with anti-VEGF siRNA or mock siRNA. As controls, x-tremeGENE/siRNA complexes were employed. Just after transfection, we created a standard scratch on the cell monolayer and recorded the number of cells that migrated into the area without cells. The percentage of intact cells that migrated was considered as 100%, while the negative control was established using the relative number of migrated cells after naked siRNA transfection. To prove the specificity of siRNA action and to prevent any effects of the carriers’ inherent toxicity, a comparison was made between the alterations in cell migration following the delivery of anti-VEGF siRNA and mock siRNA. Visual appearance of migrated EA.Hy926 cells after transfection is shown in Figure S2.

Figure 6 shows that both the negative control and RGD0-R6-complexes did not have an impact on the migration of endothelial cells. By contrast, the number of migrated cells was reduced by approximately two-fold when using anti-VEGF siRNA/x-tremeGENE complexes. However, a similar decrease in cell migration was observed also after x-tremeGENE-mediated delivery of mock siRNA. This finding clearly indicates that x-tremeGENE has a detrimental impact on endothelial cells. The RGD1-R6/siRNA complexes mainly hindered the movement of endothelial cells due to the targeted effects of the anti-VEGF siRNA (Figure 6). The inhibition efficiency of RGD1-R6 complexes formed at N/P ratios of 8/1 and 16/1 showed a marked difference between anti-VEGF-siRNA and mock siRNA complexes. Figure 6 displays a reduction in cell migration up to 25.9% and 22.6%, respectively. Thus, the effective inhibition of endothelial cell migration can be achieved by utilizing iRGD ligand-modified anti-VEGF siRNA complexes with the optimal charge ratios. As a result, these complexes demonstrate considerable potential as powerful anti-angiogenic agents that can be applied in vivo in animal model of EM.

### 3.6. Anti-Angiogenic Treatment of EM In Vivo by siRNA Complexes

**Figure 7:**
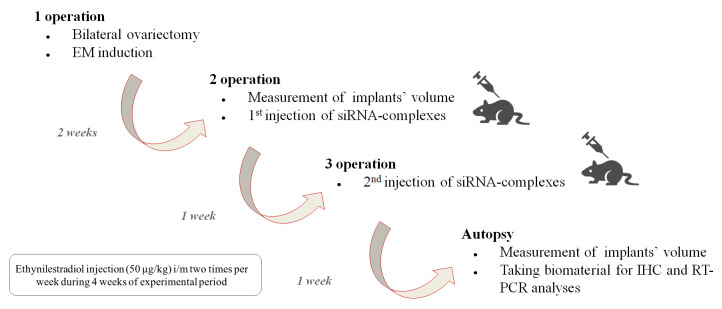
Scheme illustrating induction of endometriosis in rats and subsequent assessment of the efficiency of RGD1-R6-siRNA complexes in vivo.

To assess the efficiency of RGD1-R6/siRNA complexes in EM gene therapy, we established an animal model in Wistar rats via the autotransplantation of two fragments from the uterus horn subcutaneously. The subcutaneous implantation technique is more rewarding than intraperitoneal implantation because it allows for easy observation of endometriotic lesions that can be treated in situ [30]. In order to standardize the estrogen background among all animals, an ovariectomy was performed to synchronize the estrous cycle in the rat model. Considering that EM is a disease that depends on estrogen level, we supplemented ovariectomized animals with ethinylestradiol for two weeks prior to injecting siRNA/peptide polyplexes into the lesions. The supplementation continued for two weeks after the injection (Figure 7).

The most effective in vitro formulation of siRNA and RGD1-R6 carrier with an N/P ratio of 1/16 was tested in vivo. The anti-angiogenic therapeutic effects of RGD1-R6-mediated delivery of anti-VEGFA siRNA were assessed, comparing it with the delivery of mock siRNA using the same carrier and with an intact contralateral implant in the same animal. Saline injections were employed as a negative control, whereas Dienogest treatment per os was used as a positive control. Dienogest is an oral progestin approved for endometriosis treatment in many countries and is recommended as a first-line therapy drug [44].

**Figure 8:**
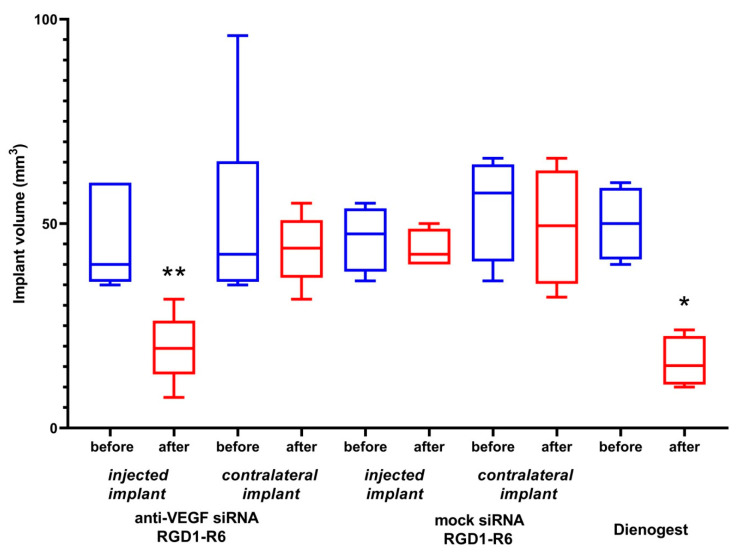
Volume of endometriotic implants before and after the treatment with the siRNA complexes. * *p* < 0.05, ** *p* < 0.01 when compared with untreated implants. The data are shown as the median ± min/max value of n = 4–6 animals per experimental group.

Each rat underwent three operations. EM was induced during the initial surgical procedure. Weight of the animals was tracked during the experimental period (Figure S3). All experimental animals developed smooth bulging endometriotic lesions by day 14 after implantation. The measurement of the endometriotic implants’ volume and the injection of the complexes were performed after 2 and 3 weeks of ethinylestradiol treatment, respectively, during the second and third operations. In total, two injections were performed with overall siRNA dose 10 µg. Figure 8 illustrates changes in volumes of endometriotic implants before and after the treatment by siRNA complexes. Significant reduction in the implants volumes was registered in anti-VEGFA siRNA treated animals compared to the mock siRNA control (*p* < 0.01). Intact contralateral implants did not change their size, whereas implants’ volume after treatment with Dienogest decreased approximately two-fold. Similar efficiency was registered after RGD1-R6-mediated delivery of anti-VEGFA siRNA (Figure 8). The obtained results on reduction in endometriotic implants growth can be compared favorably with other studies on animal EM models devoted to CXCR4-targeted delivery of anti-VEGFA siRNA and CD44-targeted delivery of anti-AQP2 siRNA [17,18]. Important to note is that the efficiency of Dienogest is comparable to that of anti-VEGF siRNA/RGD1-R6 treatment. No significant differences were found. Today, Dienogest stands as the forefront and most effective medication for the hormonal treatment of endometriosis. Reports have shown that it has the ability to alleviate pain and decrease the likelihood of disease returning [44]. However, there are indeed adverse effects, such as weight gain, elevated blood pressure, breast tenderness, and nausea. One more drawback of EM hormonal therapy is its dependence on long-term usage, which often leads to a high rate of recurrence once the treatment is stopped [45]. Thus, alternative approaches or supplementary therapies should be sought out for individuals who are unresponsive or experience unacceptable side effects from hormonal treatment.

**Figure 9:**
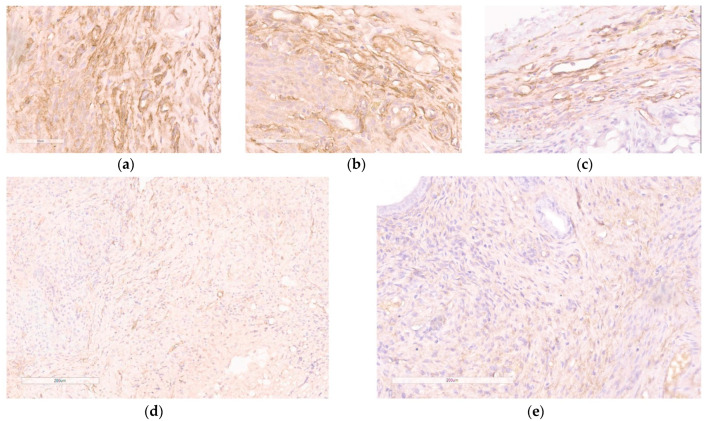
Immunohistochemical detection of CD34 expression in paraffin sections of endometrial implants after the treatment with the siRNA complexes: (**a**) intact contralateral implant; (**b**) implant injected with saline; (**c**) implant injected with RGD1-R6/mock siRNA complexes (magnification 400×, bar represents 50 µm); (**d**) implant injected with RGD1-R6/anti-VEGF siRNA complexes; (**e**) implant of rat treated with Dienogest (magnification 200×, bar represents 200 µm).

Various studies focusing on EM gene therapy have previously shown a decrease in the number of microvessels within endometriotic lesions [17,18,46,47,48]. We employed immunostaining for CD34, a marker of endothelial cells, to identify the emerging microvessels that facilitate the growth of endometriotic lesions. CD34-positive microvessels were found in the endometriotic stroma and were plentiful in the contralateral implants as well as the implants injected with saline or mock siRNA (Figure 9a–c). However, when the anti-VEGFA siRNA was delivered, there was a significant reduction in CD34-immunostaining within the injected endometriotic implants (Figure 9d). A similar pattern was shown for Dienogest-treated animals (Figure 9e). Significant differences were observed in the detection of microvessels within endometriotic lesions when treated with anti-VEGFA siRNA compared to mock siRNA complexes, which confirms the RNAi-based therapeutic anti-angiogenic effect of the therapy (Figure 9c,d).

**Figure 10:**
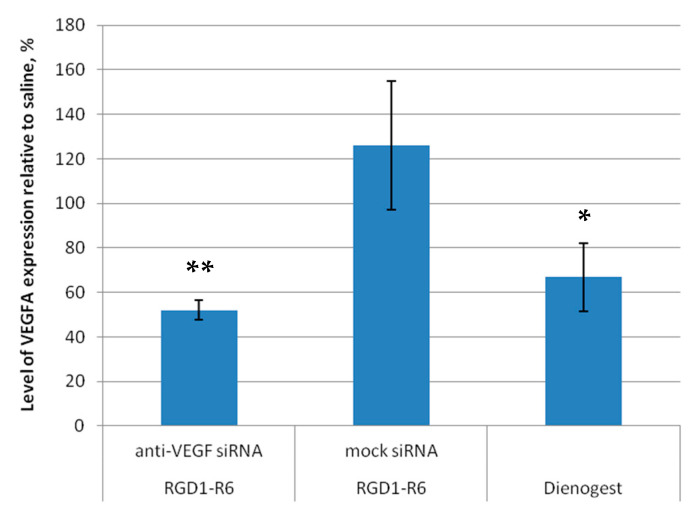
VEGFA gene expression level of endometriotic implants before and after the treatment with the siRNA complexes relative to baseline level in saline injected implants. * *p* < 0.05, ** *p* < 0.01 when compared with saline injected implants, taken as baseline (100%). The data are shown as the mean ± S.E.M.

Further, evaluation of efficiency of the RNAi-based therapy involved analyzing the target gene expression in endometrial implants before and after anti-VEGF siRNA delivery. The VEGFA gene expression in saline-injected implants was taken as a baseline (100%). Our findings demonstrate that administering anti-VEGFA siRNA using the RGD1-R6 carrier resulted in a significant decrease in VEGFA gene expression levels, up to 52.1 ± 4.5% compared to baseline values (Figure 10). As a result, there was a noticeable two-fold decrease (*p* > 0.01) in the expression of VEGFA transcripts in the treated lesions when compared to the control saline injected implants. Worth noting is that administration of complexes containing mock siRNA did not lead to a decrease in VEGFA expression. The expression levels of VEGFA were elevated above the baseline values and recorded at 126.1 ± 28.8%. Thus, the quantitative gene expression analysis revealed a specific RNAi-mediated down-regulation of VEGFA gene expression. The use of Dienogest led to a substantial decrease in the expression of the VEGFA gene, reaching up to 66.8 ± 15.2% as shown in Figure 9. Previously, it was demonstrated that Dienogest exhibits anti-angiogenic activity; however, the mechanism of its action is not clarified so far [49]. In different studies on EM animal models, contradictory data on Dienogest-mediated VEGF gene expression down-regulation were presented [50,51,52]. Herein, we show that one month of Dienogest treatment can decrease VEGFA gene expression by one third from baseline level, whereas RNAi-based therapy has a slightly better effect.

The employment of RNAi to target pathological angiogenesis is a very promising and much-needed strategy to combat angiogenesis-related illnesses caused by uncontrolled neovascularization, including but not limited to cancer, atherosclerosis, endometriosis, and age-related macular degeneration, among others. Nevertheless, the development of efficient and specific siRNA delivery systems remains a crucial necessity for the advancement of this highly promising approach. Several studies have investigated the effectiveness of gene therapy in treating EM using non-viral delivery. Zhao et al. exploited potent anti-angiogenic activity of plasmid-encoded pigment epithelium-derived factor delivered by stearic acid-grafted chitosan oligosaccharide micelles (CSO-SA/PEDF). They achieved an almost-two-fold decrease in the size of endometriotic implants in a rat EM model [46]. Later, the same group demonstrated a similar efficiency of EM treatment after CD44-targeted delivery of siRNA against aquaporin 2 [17]. Another study was devoted to anti-angiogenic gene therapy using an endostatin-encoding plasmid delivered with a PAMAM dendrimer to a mouse EM model. They show that after 30 days of this treatment the size of the implants reduced by 2–3 folds [48]. An alternate approach was reported by Liang et al., who applied anti-miRNA suppression. In this study, miR-200c mimic RNA was delivered in situ with PEI–PEG–RGD nanoparticles to endometriotic implants that resulted in approximately 1.5-fold decrease in their volume [53]. The latest study on EM gene therapy described the use of non-viral CD44-targeted pDNA delivery to modulate expression of autophagy-related Beclin-1 gene in an EM mouse model. The authors reached an almost-5-fold decrease in EM lesions volume [54]. It can be concluded, that use of pDNA and siRNA nanoparticles in the treatment of EM is a promising area despite being recent, with an increasing number of studies pointing out nanomedicine’s potential for innovative therapeutic approaches to combat this debilitating condition.

## 4. Conclusions

Overall, our findings validate the capacity of the RGD1-R6 peptide-based carrier to effectively deliver anti-VEGFA siRNA to endothelial cells in vitro and to endometriotic lesions in vivo. The presented study shows that the administration of anti-VEGFA siRNA led to a decrease in VEGFA gene expression and causing anti-angiogenic effects that resulted in the reduction in the size of endometrial implants. We demonstrate the effective endometriotic tissue penetration by RGD1-R6-siRNA complexes, following a local intra-implant injection. However, to ensure RGD1-R6 carrier future success, it is important to investigate its potential to overcome other extracellular barriers in vivo. This involves the exploration of its circulation stability, as well as the development of strategies to hinder opsonization and macrophage uptake.

## Data Availability

The data presented in this study are available on request from the corresponding author.

## Supplementary Materials

The following supporting information can be downloaded at: https://www.mdpi.com/article/10.3390/pharmaceutics15082108/s1, Figure S1: Silencing of GFP expression after treatment of MDA-MB-231 cells with the siRNA-complexes in presence of 10% fetal bovine serum; Figure S2: Visual appearance of migrated EA.Hy926 cells after treatment with the siRNA complexes; Figure S3: Monitoring of animals’ weights during experiment in vivo.
