# Supplementary material for: iRGD-Targeted Peptide Nanoparticles for Anti-Angiogenic RNAi-Based Therapy of Endometriosis
Source: Pharmaceutics. 2023 Aug 9;15(8):2108. doi: 10.3390/pharmaceutics15082108 (PMC10459007; doi:10.3390/pharmaceutics15082108)
Supplement: Supplementary file 1 [file pharmaceutics-15-02108-s001.zip › pharmaceutics-2509282-supplementary.pdf]

Supporting Information for the Article

iRGD-targeted peptide nanoparticles for anti-angiogenic RNAi-based therapy of endometriosis

Anna Egorova[1], Mariya Petrosyan[2†], Marianna Maretina[1], Elena Bazian[2], Iuliia Krylova[3], Vladislav Baranov[1†] and Anton Kiselev[1,*]

[1] Department of Genomic Medicine, D.O. Ott Research Institute of Obstetrics, Gynecology and Reproductology,Mendeleevskaya Line 3, 199034 Saint-Petersburg, Russia; A.E. egorova_anna@yahoo.com;

[2] Pharmacology Group, D.O. Ott Research Institute of Obstetrics, Gynecology and Reproductology, Mendeleevskaya Line 3, 199034 Saint-Petersburg, Russia

[3] Department of Pathology, Pavlov First Saint-Petersburg State Medical University, L'va Tolstogo Street 6-8, 197022 Saint-Petersburg, Russia

* Correspondence: ankiselev@yahoo.co.uk

† This work is dedicated to memory of our colleagues who passed away in 2022 year

Contents

Figure S1. Silencing of GFP expression after treatment of MDA-MB-231 cells with the siRNA-complexes in presence of 10% fetal bovine serum………....................................3

Figure S2. Visual appearance of migrated EA.Hy926 cells after treatment with the siRNA complexes……………………..4

Figure S3. Monitoring of animals' weights during experiment in vivo 6

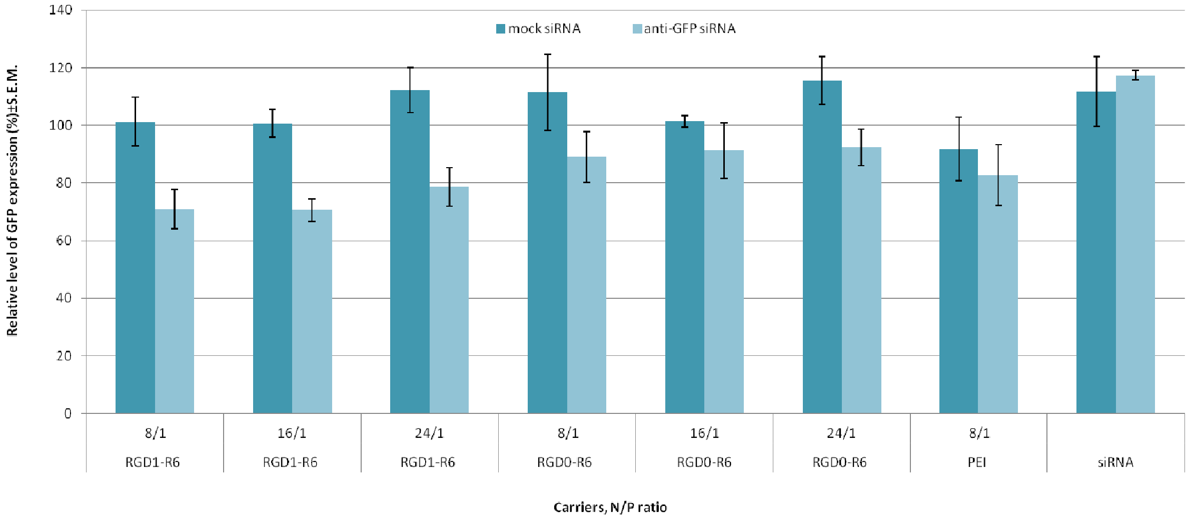

Figure S1. Silencing of GFP expression after treatment of MDA-MB-231 cells with the siRNA-complexes in presence of 10% fetal bovine serum. * - $p<0.05$ when compared with cells treated by corresponding mock siRNA-complexes. The data are shown as the mean ± S.E.M.

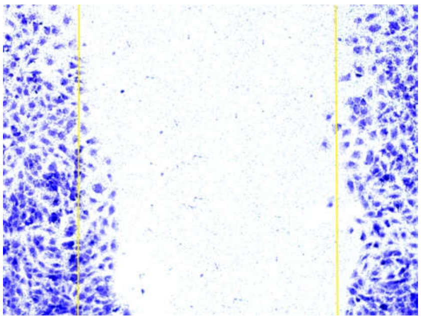 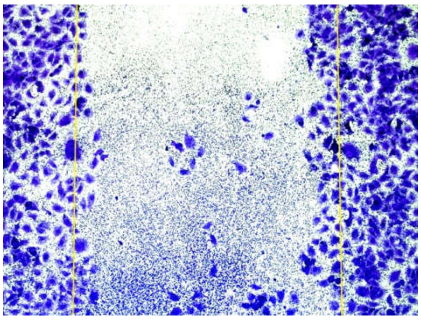

(a) (b)

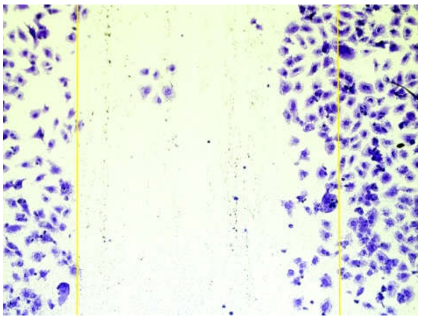 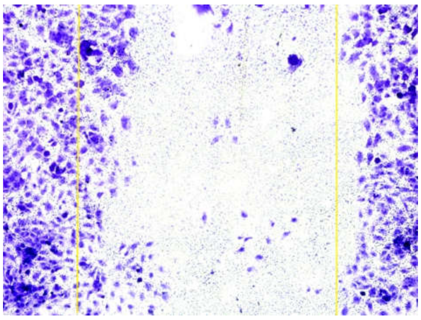

(c) (d)

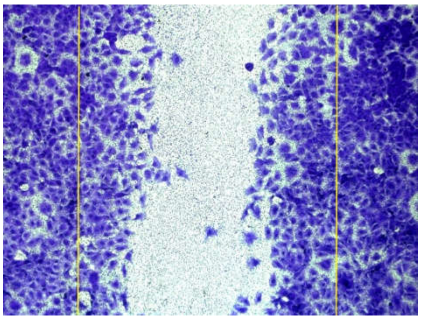 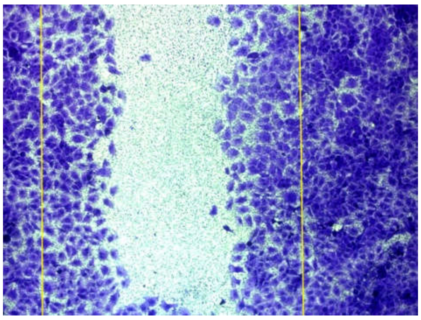

(e) (f)

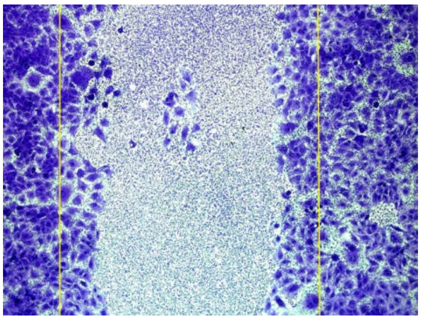 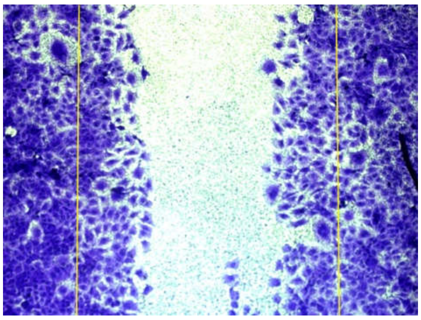

(g) (h)

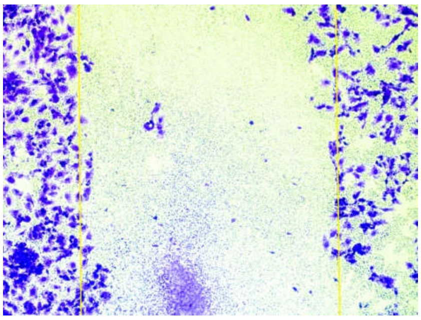 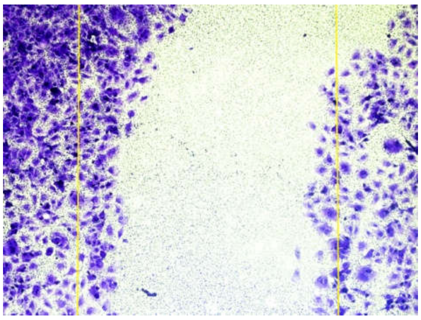

(i) (j)

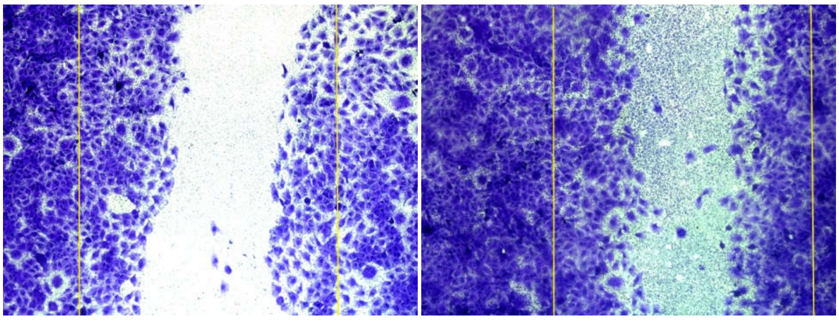

(k) (l)

Figure S2. Visual appearance of migrated EA.Hy926 cells after treatment with **(a)** RGD1-R6/anti-VEGFA siRNA at N/P ratio 8/1 **(b)** RGD1-R6/mock siRNA at N/P ratio 8/1, **(c)** RGD1-R6/anti-VEGFA at N/P ratio 16/1, **(d)** RGD1-R6/mock siRNA at N/P ratio 16/1, **(e)** RGD0-R6/anti-VEGFA at N/P ratio 8/1, **(f)** RGD0-R6/mock siRNA at N/P ratio 8/1, **(g)** RGD0-R6/anti-VEGFA at N/P ratio 16/1, **(h)** RGD0-R6/mock siRNA at N/P ratio 16/1, **(i)** x-tremeGENE/anti-VEGFA siRNA, **(j)** x-tremeGENE/mock siRNA, **(k)** naked anti-VEGFA siRNA, **(l)** mock siRNA.

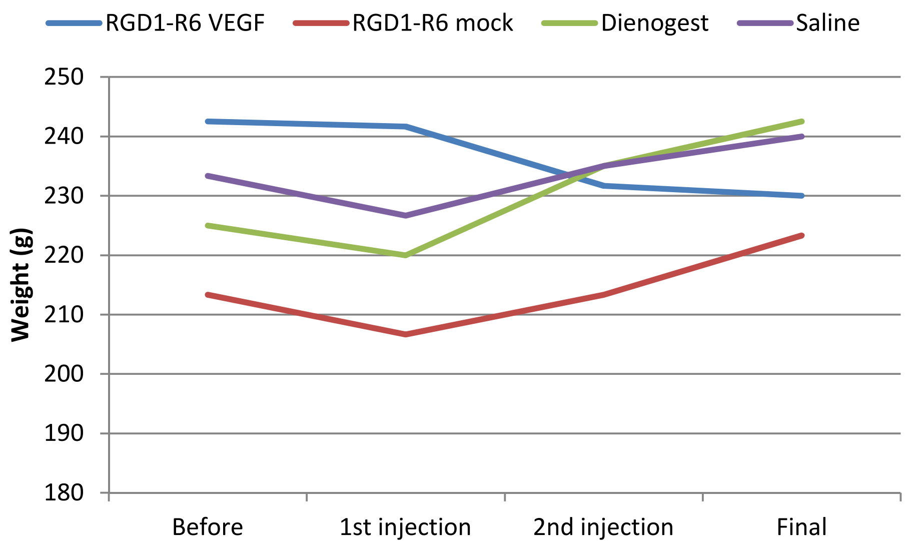

Figure S3. Monitoring of animals' weights during experiment in vivo.
